# Evaluating the efficacy and safety of bromhexine hydrochloride tablets in treating pediatric COVID-19: A protocol for meta-analysis and systematic review

**DOI:** 10.1097/MD.0000000000022114

**Published:** 2020-09-11

**Authors:** Yuying Wang, Yinghua Zhang, Xia Chen, Kun Xue, Tianjing Zhang, Xiaohong Ren

**Affiliations:** a Department of Pediatric, Baoji Maternal and Child Health Hospital, Baoji; b Department of Pediatric, Taian Maternal and Child Health Hospital, Taian; c The Fifth Department of Pediatric, Baoji Maternal and Child Health Hospital, Baoji; d Department of Pediatric, Peking Union Medical College Hospital, Beijing, China.

**Keywords:** bromhexine hydrochloride tablets, children, COVID-19, meta-analysis, protocol

## Abstract

**Background:**

Bromhexine hydrochloride tablets may be effective in the treatment of Coronavirus disease 2019 (COVID-19) in children. This study will further evaluate the efficacy and safety of bromhexine hydrochloride tablets in the treatment of COVID-19 in children.

**Methods:**

The following electronic databases will be searched, with all relevant randomized controlled trials (RCTs) up to August 2020 to be included: PubMed, Embase, Web of Science, the Cochrane Library, China National Knowledge Infrastructure (CNKI), the Chongqing VIP China Science and Technology Database (VIP), Wanfang, the Technology Periodical Database, and the Chinese Biomedical Literature Database (CBM). As well as the above, Baidu, the International Clinical Trials Registry Platform (ICTRP), Google Scholar, and the Chinese Clinical Trial Registry (ChiCTR) will also be searched to obtain more comprehensive data. Besides, the references of the included literature will also be traced to supplement our search results and to obtain all relevant literature.

**Results:**

This systematic review will evaluate the current status of bromhexine hydrochloride in the treatment of COVID-19 in children, to evaluate its efficacy and safety.

**Conclusion:**

This study will provide the latest evidence for evaluating the efficacy and safety of bromhexine hydrochloride in the treatment of COVID-19 in children.

**PROSPERO Registration number:**

CRD42020199805.

**Ethics and dissemination:**

The private information of individuals will not be published. This systematic review will also not involve endangering participant rights. Ethical approval is not available. The results may be published in peer-reviewed journals or disseminated at relevant conferences.

## 1 Introduction

Novel coronavirus (SARS-CoV-2) can be transmitted through droplets, close contact, aerosols, and other forms.^[1]^ People gathering in crowds are particularly susceptible. Fever, fatigue, and dry cough are the most common clinical symptoms of COVID-19.^[2]^ At present, it is recommended to use a-interferon, lopinavir/ritonavir, ribavirin, chloroquine, and abidol for antiviral therapy.^[3–6]^ Bromhexine is only used as an adjuvant drug for symptomatic treatment to alleviate the respiratory problems of patients. Literature research and molecular simulation have found that bromhexine may have antiviral potential that has never been discovered before.^[7–9]^ Combined with the other respiratory effects of bromhexine, it may be used as a first-line drug in the clinical treatment of novel coronavirus.

SARS-CoV-2 mainly relies on transmembrane protease, serine 2 (TMPRSS2) to enter the cell.^[10,11]^ Therefore, TMPRSS2 inhibitors can be used to block SARS-CoV-2 infection.^[12]^ As early as 2014, some research teams selected bromhexine as the drug with the strongest inhibitory effect on TMPRSS2.^[13]^ It is suggested that the TMPRSS2 inhibitor bromhexine can be used to block pulmonary virus infection, whereas chloroquine can be used to inhibit the viral lysosome pathway. A large number of sticky secretions have been seen in the autopsy lung sections of COVID-19 patients who have died.^[14]^ Bromhexine can reduce sputum viscosity, promote lung ventilation and expectoration, and remove virus residues.^[15]^

At present, many clinical trials have been carried out to study the efficacy of bromhexine in the treatment of COVID-19 patients. However, the strength of the evidence for the clinical efficacy and safety of bromhexine hydrochloride tablets in the treatment of COVID-19 in children is unclear. Therefore, the purpose of this study is to evaluate the clinical efficacy and safety of bromhexine hydrochloride tablets in the treatment of COVID-19 in children.

## 2 Methods

We will conduct this work following the Preferred Reporting Items for Systematic Reviews and Meta-Analysis Protocols (PRISMA-P) statement guidelines.^[16]^ This work has been registered at PROSPERO. The registration number for this study is CRD42020199805.

### 2.1 Inclusion criteria for study selection

#### 2.1.1 Types of studies

All randomized controlled trials (RCTs) of bromhexine hydrochloride tablet treatment for COVID-19 in children will be included without language restriction. Observational studies, conference abstracts, animal studies, data which are incomplete or contain obvious errors, and letters will all be excluded.

#### 2.1.2 Types of participants

Inclusion:

1. Aged between 2 and 18 years, male or female;
2. Laboratory (reverse transcription-polymerase chain reaction) and clinical symptom-confirmed cases of COVID-19, according to the official guidelines of the “Pneumonia Diagnosis and Treatment Scheme for Novel Coronavirus Infection (Trial Version 6),” or patients with negative 2019-nCoV RNA results but suspected clinical symptoms;
3. The legal guardian of the enrolled children has given informed consent voluntarily (subjects ≥8 years’ old have also given informed consent voluntarily).

Exclusion:

1. Patients diagnosed with severe or critical pneumonia caused by novel coronavirus infection;
2. Patients allergic to the ingredients of bromhexine hydrochloride tablets and/or with definite contraindications on the label of bromhexine hydrochloride tablets;
3. Patients with a history of serious cardiovascular and cerebrovascular diseases, such as heart failure, congenital heart disease, serious arrhythmia, and cerebrovascular accident;
4. Patients with severe liver disease or liver insufficiencies, such as cirrhosis or chronic active hepatitis;
5. Patients with severe kidney disease (such as chronic renal insufficiency);
6. Patients with a history of malignant tumors;
7. Patients with serious hematopoietic diseases or agranulocytosis.

#### 2.1.3 Types of interventions

##### 2.1.3.1 Experimental interventions

Bromhexine hydrochloride tablets + standard treatment.

##### 2.1.3.2 Control interventions

Standard treatment.

#### 2.1.4 Types of outcome measures

##### 2.1.4.1 Primary outcomes

1. Rate of aggravation.
2. Clinical remission rate.
3. Rate of patients with fever.
4. Rate of patients with dyspnea.
5. Rate of patients with mechanical ventilation.
6. Rate of ICU admission.

##### 2.1.4.2 Additional outcomes

Adverse events.

### 2.2 Search methods for the identification of studies

#### 2.2.1 Electronic searches

We will search PubMed, Embase, Web of Science, the Cochrane Library, China National Knowledge Infrastructure (CNKI), the Chongqing VIP Chinese Science and Technology Database (VIP), Wanfang, the Technology Periodical Database, and the China Biomedical Literature Database (CBM) for all dates up to August 2020. The PubMed search strategy is shown in Table 1.

**Table 1:**
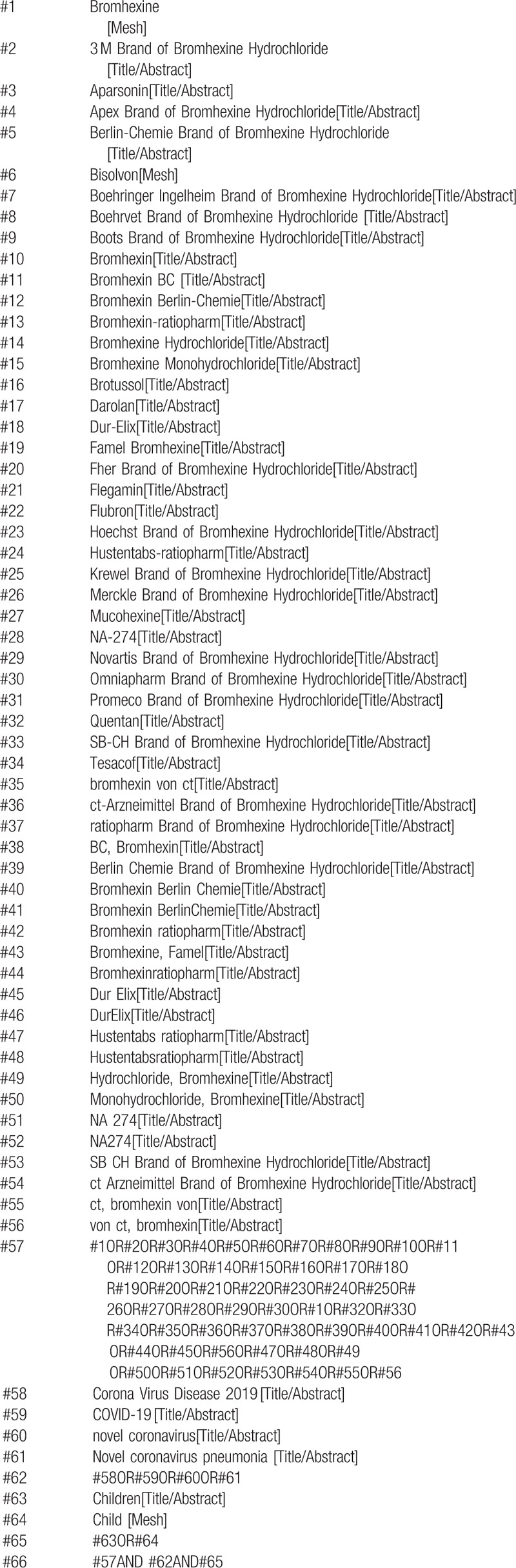
Search strategy in PubMed database.

#### 2.2.2 Search for other resources

Baidu, the International Clinical Trials Registry Platform (ICTRP), Google Scholar, and the Chinese Clinical Trial Registry (ChiCTR) will also be searched to obtain more comprehensive data. In addition, the references of the included literature will also be traced to supplement our search results and to obtain all the relevant literature.

### 2.3 Data collection and analysis

#### 2.3.1 Selection of studies

**Figure 1:**
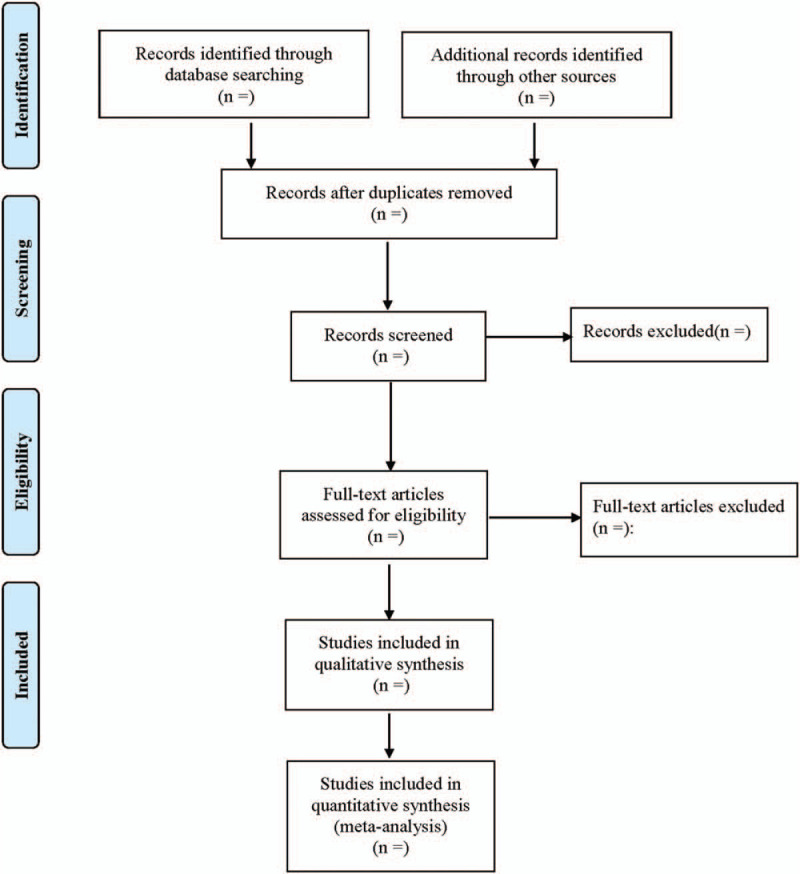
Flow diagram of study selection process.

EndNote X8 will be used for document management. Two researchers will independently sift through the literature, extract data, and cross-check their accuracy. In the aspect of literature screening, the title and abstract of the research retrieved in the retrieval process will first be read, then texts identified as related research will be read in full. Agreement must be reached between the 2 researchers to decide whether to include a text or not, with obviously irrelevant literature immediately excluded. If there is any objection, a third researcher will be consulted to assist in making the judgment. Excel 2019 will be used to create a data extraction table and extract related data. The study selection procedure is shown in Figure 1.

#### 2.3.2 Data extraction and management

The 2 reviewers will independently extract relevant data from eligible studies, and any objections will be resolved through discussion with the third reviewer. The extracted research data will mainly include the first author, year of publication, study location, baseline characteristics of participants, sample size, intervention time, follow-up, outcome measurement indicators, and adverse events. If necessary, the trial author will be contacted for more information.

### 2.4 Risk of bias assessment

The 2 researchers will independently assess the risk of bias in the inclusion of studies from the Cochrane Library according to the guidelines of the RCTs intervention system evaluation manual. The following items will be considered: random sequence generation, allocation hiding, the blindness of participants and personnel, the blindness of result evaluation, incomplete result data, selective reporting, and other sources of bias. The quality of the study will be classified as “high,” “unclear” or “low” bias risk. If there are any objections, the third researcher will be consulted and a final decision made as required.

### 2.5 Quantitative data synthesis and statistical methods

#### 2.5.1 Quantitative data synthesis

The RevMan 5.3 software (provided by the Cochrane Collaboration) will be used for the statistical analysis. For dichotomous variables, relative risk has been selected for use, and all will be represented by effect value and 95% confidence intervals.

#### 2.5.2 Assessment of heterogeneity

The Q test will be used to qualitatively determine inter-study heterogeneity, and if *P* ≥ .1, it will indicate that there is no inter-study heterogeneity. However, if *P* < .1, it will indicate that there is inter-study heterogeneity present. At the same time, the *I*^2^ statistic will be used to quantitatively evaluate the inter-study heterogeneity. If *I*^2^ ≤ 50%, heterogeneity will be considered to be low, and the fixed-effect model will be adopted for use. Conversely, if *I*^2^ > 50%, it will indicate that there is significant heterogeneity, in which case the source of the heterogeneity will be explored through a subgroup or sensitivity analysis. If there proves to be no obvious clinical or methodological heterogeneity, then it will be considered as statistical heterogeneity, and the random-effects model will then be used for the analysis instead. Descriptive analysis will be used if there is significant clinical heterogeneity between the 2 groups and subgroup analysis is not available.

#### 2.5.3 Assessment of reporting biases

Publication bias will be estimated by a funnel plot analysis if sufficient studies are included (>10 studies).

#### 2.5.4 Subgroup analysis

Subgroup analyses will be carried out based on drug dose and duration.

#### 2.5.5 Sensitivity analysis

To test the stability of the results, we will use the one by one elimination method for sensitivity analysis.

#### 2.5.6 Ethics and dissemination

This systematic review will not require ethical approval because there will be no data used in our study that is linked to individual patients. The results will be disseminated only in peer-reviewed publications.

## 3 Discussion

Bromhexine is a kind of expectorant, which is mainly used in the treatment of chronic bronchitis, emphysema, bronchiectasis, and chronic obstructive pulmonary disease.^[17–19]^ As a potent inhibitor of TMPRSS2, a key protease in the infection and transmission of novel coronavirus SARS-CoV-2, bromhexine has the advantage of low price and greater safety.^[20]^ Also, bromhexine and its metabolites can competitively bind to cellular receptor angiotensin-converting enzyme 2 (ACE2). This strongly inhibits the key M proteases of novel coronavirus SARS-Cov-2, promotes the release of endogenous active substances in the lungs, maintains alveolar function, and promotes sputum excretion.^[9]^

Related studies have pointed out that effective drugs to prevent novel coronavirus infection must contain either TMPRSS2 inhibitors or competitive ACE2 binding inhibitors, and it is particularly recommended that bromhexine, a specific TMPRSS2 inhibitor, be used to prevent and treat COVID-19.^[21]^ In addition, several university and institutional experts have jointly published articles to evaluate the importance of TMPRSS2 for respiratory virus infection and introduce the therapeutic potential of bromhexine as a TMPRSS2 inhibitor for COVID-19.^[20]^

To our knowledge, this is the first systematic review of the clinical efficacy and safety of bromhexine hydrochloride tablets in the treatment of COVID-19 in children. We believe that this review will provide evidence-based advice for a COVID-19 treatment system in children, optimize clinicians’ treatment strategies, and identify promising treatment options for researchers.

## Author contributions

**Conceptualization:** Yuying Wang and Xiaohong Ren

**Data collection:** Yinghua Zhang and Xia Chen

**Funding support:** Xiaohong Ren

**Literature retrieval:** Kun Xu and Tianjing Zhang

**Software operating:** Kun Xu and Tianjing Zhang

**Supervision:** Kun Xu and Tianjing Zhang

**Writing – original draft:** Yuying Wang and Xiaohong Ren

**Writing – review & editing:** Yuying Wang and Xiaohong Ren
